# The TNF-Family Cytokine TL1A Inhibits Proliferation of Human Activated B Cells

**DOI:** 10.1371/journal.pone.0060136

**Published:** 2013-04-02

**Authors:** Chiara Cavallini, Ornella Lovato, Anna Bertolaso, Luciano Pacelli, Elisa Zoratti, Elisabetta Zanolin, Mauro Krampera, Alberto Zamò, Cristina Tecchio, Marco A. Cassatella, Giovanni Pizzolo, Maria T. Scupoli

**Affiliations:** 1 Department of Medicine, Section of Hematology, University of Verona, Verona, Italy; 2 Interdepartmental Laboratory for Medical Research (LURM), University of Verona, Verona, Italy; 3 Department of Pathology and Diagnostics, Section of Pathological Anatomy, University of Verona, Verona, Italy; 4 Applied Research on Cancer-Network (ARC-NET), University of Verona, Verona, Italy; 5 Department of Medicine and Public Health, Section of Epidemiology and Medical Statistics, University of Verona, Verona, Italy; 6 Department of Pathology and Diagnostics, Section of General Pathology, University of Verona, Verona, Italy; Università degli Studi di Milano, Italy

## Abstract

Death receptor (DR3) 3 is a member of the TNFR superfamily. Its ligand is TNF-like ligand 1A (TL1A), a member of the TNF superfamily. TL1A/DR3 interactions have been reported to modulate the functions of T cells, NK, and NKT cells and play a crucial role in driving inflammatory processes in several T-cell-dependent autoimmune diseases. However, TL1A expression and effects on B cells remain largely unknown. In this study, we described for the first time that B cells from human blood express significant amounts of DR3 in response to B cell receptor polyclonal stimulation. The relevance of these results has been confirmed by immunofluorescence analysis in tonsil and spleen tissue specimens, which showed the *in situ* expression of DR3 in antigen-stimulated B cells *in vivo*. Remarkably, we demonstrated that TL1A reduces B-cell proliferation induced by anti-IgM-antibodies and IL-2 but did not affect B-cell survival, suggesting that TL1A inhibits the signal(s) important for B-cell proliferation. These results revealed a novel function of TL1A in modulating B-cell proliferation *in vitro* and suggest that TL1A may contribute to homeostasis of effector B-cell functions in immune response and host defense, thus supporting the role of the TL1A/DR3 functional axis in modulating the adaptive immune response.

## Introduction

Death receptor (DR) 3 (TNFRSF25/Apo3/LARD/TR3/TRAMP/WSL-1) is a member of the TNFR superfamily and, within that family, of the DR subfamily, whose members contain a death domain (DD) as part of their intracellular domain [1]–[5]. Among the DR subfamily members, DR3 shows the highest homology to TNFR1 [3], [4]. However, unlike TNFR1 that shows a ubiquitous expression, DR3 expression is restricted to lymphocyte-enriched tissues, including peripheral blood leukocytes, thymus and spleen, and it has been shown to be especially up-regulated in activated T cells [2], [6].

The ligand for DR3 is TNF-like ligand 1A (TL1A), a member of the TNF superfamily [7]–[10]. TL1A is expressed in a variety of cell types, including activated endothelial cells, monocytes, macrophages, dendritic cells, and T cells [7], [11]–[15]. Like other TNF members, TL1A contains a predicted transmembrane domain and a bioactive, proteolytically cleaved truncated form that can be released as a soluble factor [7], [8]. TL1A expression is highly regulated and induced by inflammatory stimuli [7], [11], [15], [16].

The TL1A/DR3 axis has been shown to costimulate T cells to produce a wide variety of cytokines and promote cell proliferation of activated T cells *in vitro* and *in vivo* [11], [13]. TL1A biases T cells to differentiate towards Th1 and Th17 phenotype [12], [13], [17] and modulates Treg expansion and functions [18]–[20]. Moreover, DR3 can modulate NK [6], [21] and NKT-cell functions [22]. Consistently, recent studies have reported an essential role for the TL1A/DR3 pathway in generating T-cell host defense response [23], [24].

Several studies in mice deficient in DR3 or TL1A have revealed a specific role for DR3 in enhancing proliferation of effector T cells at the site of tissue inflammation in autoimmune disease models driven by diverse T-cell subsets, with the level of TL1A expression correlating with the severity of inflammation [25], [26]. Moreover, chronic expression of TL1A induces a distinct interleukin-13-dependent inflammatory disease of small intestine [20]. Together, these studies have contribute to shape a model for TL1A/DR3 axis suggesting that TL1A produced by endothelial cells, dendritic cells, and monocytes in inflamed tissue provides costimulation for effector and memory T cells leading to greater pathogenicity in diverse autoimmune diseases [25], [26].

Over the last few years, major efforts have been made to understand the physiologic and pathologic role of TL1A in T cells. In contrast, little is known on expression and function of DR3 in B cells. To address this issue, in this study we explored the expression of DR3 in human B cells derived from peripheral blood and investigated its possible role in modulating B-cell proliferation. We described that B cells activated *in vitro* by the B cell receptor (BCR) stimulation express DR3 molecule. Further, DR3 was expressed *in vivo* in antigen-stimulated B cells of tonsil germinal centers (GC). Remarkably, we found that TL1A significantly reduces proliferation of suboptimally activated B cells. Our data suggest a novel role for the TL1A/DR3 axis in modulating proliferation of activated B cells.

## Materials and Methods

### Cell and Tissue Samples

Cryopreserved peripheral blood mononuclear cells (PBMC) from 10 human blood buffy coats and formalin-fixed paraffin-embedded human tissue tonsil (n = 4) and spleen (n = 3) sections were used in this study. Buffy coats were collected at the Hematology Unit, Azienda Ospedaliera Universitaria Integrata (AOUI) in Verona (Italy); tonsil specimens were obtained from hyperplastic tonsils of subjects undergoing tonsillectomy and collected at the Pathological Anatomy Unit, AOUI, Verona (Italy); spleen specimens were obtained from normal spleen removed after traumatic injuries and collected at the Pathological Anatomy Unit, AOUI, Verona (Italy). PBMCs were isolated by Ficoll-hypaque centrifugation (Lymphoprep, Nicomed, Oslo, Norway) and suspended in freezing medium for storage in liquid nitrogen. Upon thawing, cell viability consistently exceeded 95% in all samples. Cells were washed twice in PBS and then resuspended in the appropriate buffer or medium. PBMC-derived B cells were isolated by negative selection using the Human B-Cell Enrichment Kit (without CD43 depletion; Stem Cell Technologies, Vancouver, Canada). After separation, B cells were washed twice and counted. Cell purity as assessed with CD19 staining was routinely above 98%.

### Ethics Statement

Blood and tissue samples were collected under a protocol approved by the local Ethics Committee (Comitato Etico per la Sperimentazione – AOUI) and data were analyzed anonymously. In accordance with the Declaration of Helsinki, all blood donors provided written informed consent for the collection and use of their blood samples for research purposes. For the use of tissue samples, the local Ethics Committee (Comitato Etico per la Sperimentazione – AOUI) approved the anonymous retrospective use of samples consisting of “diagnostic remnants” without written consent release, as also specifically stated in the Italian law, according to the directive issued on March 1st 2012 from the Italian Privacy Authority (Deliberazione n. 85) (12A03185) (complying with EU directives).

### Cell Stimulation

Peripheral blood (PB) purified B cells were stimulated by incubating with sulfate latex beads (2.3 µm diameter) (Interfacial Dynamics Corporation, Portland, OR) [27] coated with goat F(ab’)_2_ anti-human IgM (20 µg/ml) (Southern Biotech, Birmingham, AL) in 24-well plates, at 5×10^6^ cells/ml, for the indicated time. At the end of the incubation, the cells were subjected to flow cytometry or biochemical analysis.

### Flow Cytometry Analyses

PB purified B cells stimulated or not with sulfate latex beads coated with anti-IgM for 24 h were harvested, washed, resuspended in PBS and incubated with either PE-conjugated anti-human DR3 mAb (clone JD3, BioLegend, London, UK) or isotype control antibody on ice for 45 min. The cells were then stained with APC-conjugated anti-CD19 mAb (BD Biosciences, San Jose, CA) and 7-amino-actinomycin (7AAD, BD Biosciences) for 15 min on ice. Approximately 1×10^4^ gated events were acquired for each sample on FACSCanto cytometer (Becton Dickinson, San Jose, CA). Flow cytometry data were gated using the FlowJo software (TreeStar, Ashland, OR). All analyses were gated on lymphocytes based on forward and side scatter, on living cells (7AAD-negative), and on B cells (CD19-positive). Fluorescence signals were normalized with respect to the controls by calculating the ratio between the median fluorescence intensity (MFI) of DR3 and the respective isotype-matched, irrelevant monoclonal antibody.

For immunophenotype analysis of B cells stimulated with anti-IgM, IL-2 in the presence or absence of TL1A, PB purified B cells were plated in a 96-well plate at 1×10^5^/well in duplicate and stimulated with 2 µg/ml of soluble anti-IgM, 20 U/ml IL-2 in presence or absence of 100 ng/ml human recombinant TL1A. Cells were harvested at 24, 48, 72 and 96 h and stained with 7AAD, FITC-conjugated anti-CD38, PE-conjugated anti-CD20, PECy7-conjugated anti-CD19, APC-conjugated anti-CD138, and APC-H7-conjugated anti-CD45 mAb (all from BD Biosciences). Approximately, 1×10^4^ gated events were acquired for each sample on a FACSCanto (Becton Dickinson) and analyzed using FlowJo software (TreeStar). Multiparameter phenotyping of B cells was performed gating on lymphocytes, based on forward and side scatter, on living cells according to 7AAD staining, and then on B cells on the basis of CD19 and CD45 expression. When indicated, IgM-positive or -negative (IgM+; IgM−) B cells were gated for analysis.

### Western Blot Analysis

PB purified B cells stimulated or not with sulfate latex beads coated with anti-IgM for 24 h, as described above, were lysed in 80 µl of lysis buffer (50 mM Tris [pH 7.5], 150 mM NaCl, 1 mM EDTA, 1 mM EGTA, 0.5% Igepal, 1% Triton X-100; all supplied by Sigma-Aldrich, Milan, Italy) supplemented with 1 mM Na_3_VO_4_, 1 mM PMSF and protease inhibitors (all supplied by Sigma-Aldrich). Protein concentrations were normalized using BCA reagent according to the manufacturer’s protocol (Sigma-Aldrich). Fifteen µg proteins were separated on a 10% SDS-polyacrylamide gel and electrotransferred to a PVDF membrane. The membrane was treated with blocking buffer (5% BSA in Tris buffered saline +0.1% Tween-20 [TBST]) and then incubated with anti-DR3 primary antibody (Cell Signaling Technology, Danvers, MA, USA). Blots were developed using chemiluminescent substrate Lite A Blot Plus (Euroclone S.p.A., Siziano, Italy) and horseradish peroxidase (HRP)-conjugated secondary antibodies. β-actin was used as standard. Quantification of bands was performed with ImageJ software (http://rsb.info.nih.gov/ij/). Chemiluminescent signals were normalized calculating the ratio between DR3 expression of each sample and the respective β-actin signal. Changes in DR3 expression was expressed by calculating the fold difference in normalized DR3 of anti-IgM stimulated cells divided by resting cells (i.e. fold change).

### Immunofluorescence

Staining was performed on 4 µm formalin-fixed paraffin-embedded tissue tonsil and spleen sections. Deparafinization was obtained by a 20-minute wash in xylene and hydration by sequential washes in 100%, 85%, and 70% ethanol solutions (10 min), distilled water (10 min) and twice in PBS (10 min each). Sections were bathed in ER2 solution (pH 8, Leica Biosystems, Newcastle, UK) at 85°C for 30 min for antigen retrieval and then cooled 15 min at room temperature and 15 min in water. Next, the slides were rinsed with distilled water for two minutes, and then with PBS. Incubation with each antibody was preceded by 20 min protein block (DAKO, Carpinteria, CA, USA). Slides were incubated with primary antibody in dilution solution (Leica Biosystems) (DR3, clone JD3, dil. 1∶50, Abcam, Cambridge, UK; CD20, clone L26, Leica Biosystems, dil. 1∶100; CD3, clone SP7, Leica Biosystems, dil. 1∶150) overnight at 4°C (DR3) or for 1 h at room temperature (CD20 and CD3) in a humid chamber. Anti-mouse (DR3 and CD20) or anti-rabbit (CD3) biotinilated antibody was incubated for 30 min at room temperature. Then Qdot Streptavidin Conjugates (QD565, QD605, QD655, Invitrogen, Eugene, OR, USA) were incubated in PBS (40 nM) for 1 h at RT in a humid chamber. Slides were then rinsed in PBS twice 3 min each and incubated with Qnuclear Deep Red Stain (Invitrogen; dil. 1∶1000 in PBS) for 20 min. After rinsing slides again in PBS, they were washed in graded ethanol solution series (30 s each) consisting of 30%, 50%, 70%, and 90% ethanol, and then for 1 min with Toluene (Sigma-Aldrich). Slides were mounted with Qdot Qmount mounting media (Invitrogen).

Images were acquired with microscope BX61, Olympus optical CO. LTD. (Tokyo, Japan) and with proper filter sets (QD565, QD605, QD655, Chroma Technology Corporation, Bellow Falls, VT, USA), and for nuclear staining Cy5 filter set (Olympus). Images were finally analyzed and elaborated by CellF software v. 3.3 (Olympus Soft Imaging Solutions GmbH, Munster, Germany).

### Proliferation Analysis

PB purified B cells were resuspended at 1×10^6^/ml in PBS at 37°C supplemented with 0.1% BSA and labeled with 5 µM CFSE (Invitrogen) for 10 min at 37°C. The reaction was quenched adding ice-cold RPMI 1640 medium supplemented with 10% FBS and incubating on ice for 5 min. Cells were then washed three times with ice-cold RPMI 1640 medium supplemented with 10% FBS. CFSE-labeled cells were plated in a 96-well plate at 1×10^5^/well in duplicate and stimulated with the indicated concentrations of soluble anti-IgM and 20 U/ml IL-2 (Proleukin, Novartis, Siena, Italy), 2.5 µg/ml CpG oligodeoxynucleotides type B 2006-G5 (CpG-ODN, Invivogen, Toulouse, France) or 50 ng/ml polyhistidine-tagged CD40L (R&D System, Abington, UK), in the presence or absence of the indicated concentrations of human recombinant TL1A (Peprotech, London, UK). For stimulation with anti-IgM and IL-2, cells were harvested after 24, 48, 72 and 96 h and stained with 7AAD, PECy7-conjugated anti-CD19 and APC-conjugated anti-CD27 mAb (all from BD Biosciences). For stimulation with anti-IgM and CpG-ODN or CD40L, cells were harvested after 96 h and stained with 7AAD and APC-conjugated anti-CD19. Approximately, 3×10^4^ gated events were acquired for each sample on a FACSCanto (Becton Dickinson).

CFSE dilution was analyzed on B cells using FlowJo software (TreeStar) comparing three different parameters: (i) division index, i.e. the average number of divisions that a cell has undergone; (ii) percentage divided, i.e. the percentage of cells that divided in the original sample; (iii) number of cell divisions, calculated from the number of cells in each population. All analyses were gated on lymphocytes based on forward and side scatter, living cells according to 7AAD staining, B cells on the basis of CD19 expression, and discriminating CD27-positive (CD27+) and −negative (CD27−) cells.

### Apoptosis Assay

Cell apoptosis was detected using the annexinV-FITC/propidium iodide (PI) detection kit (Bender Med System, Vienna, Austria), according to the manufacturer’s recommended protocol. Briefly, purified B cells were stimulated with 2 µg/ml soluble anti-IgM, 20 U/ml IL-2 (Proleukin, Novartis) in the presence or absence of 100 ng/ml human recombinant TL1A (Peprotech). After 24, 48, 72 and 96 h, the cells were harvested and stained with FITC-conjugated annexinV for 10 min. PI was added just before the analysis on a FACSCanto flow cytometer (Becton Dickinson). At least 1×10^4^ events were acquired. Samples were analyzed by FlowJo software (TreeStar). Viable cells were defined as annexinV-negative and PI-negative. All analyses were gated on CD19 expression.

### Statistical Analysis

All graphing and statistical analyses were performed using GraphPad Prism software. Results obtained in independent experiments are expressed as mean and SEM. As data were not normally distributed, the two-sample Wilcoxon signed rank sum test was used to compare DR3 expression between resting and anti-IgM-stimulated cells and compare B cell proliferation between samples subjected to different stimuli. The Mann Whitney test was used to compare DR3 expression between IgM-positive and IgM-negative B cells. Student t test was used to compare proliferation between CD27-positive and CD27-negative B-cell subsets, for which two independent experiments have been performed. Two-way ANOVA was used to compare cell survival between samples subjected to different stimuli. Differences between data were considered significant for *p*-values <0.05.

## Results

### Expression of DR3 in B Cells

**Figure 1:**
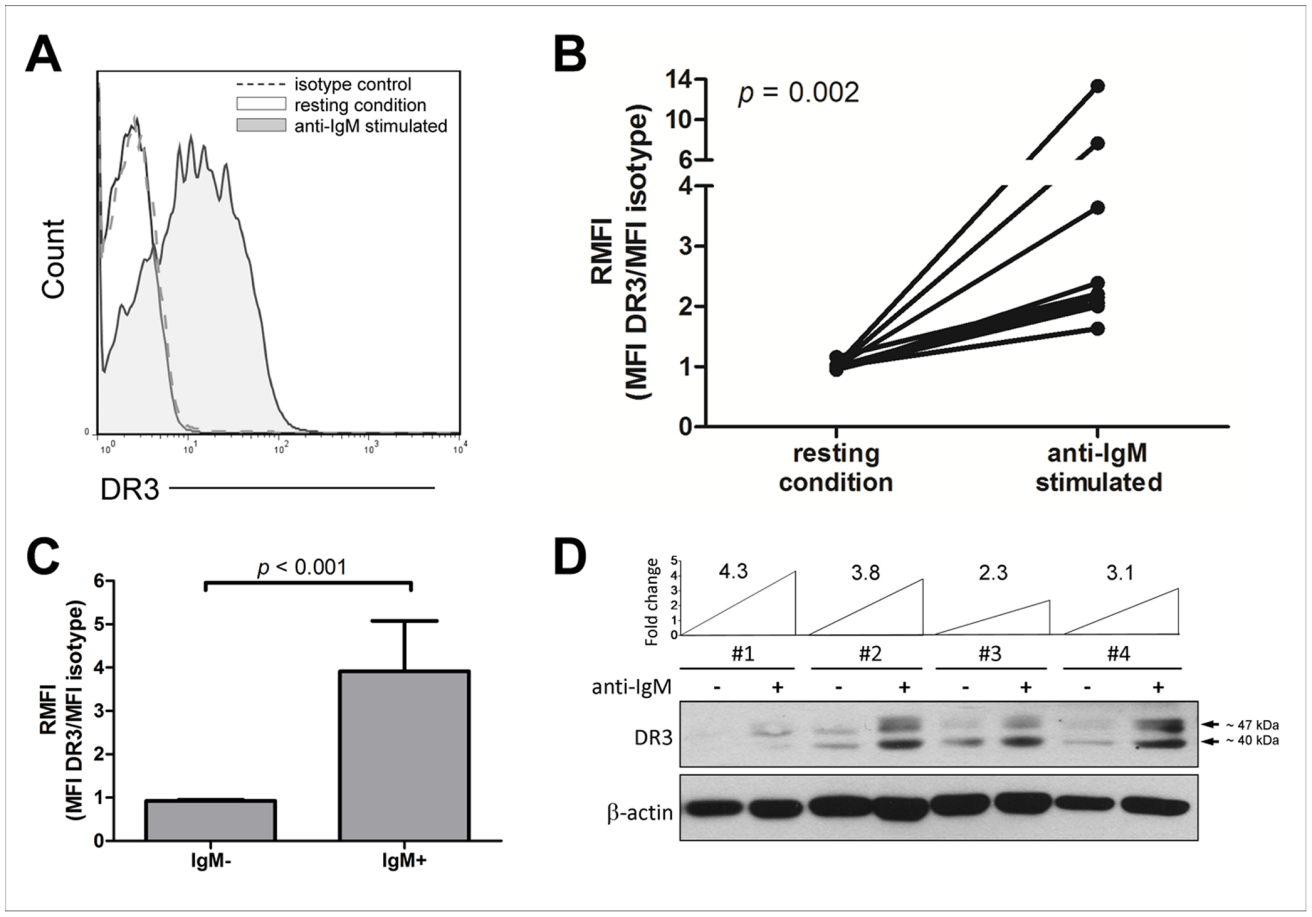
DR3 surface expression in B cells. (**A**) Representative flow cytometry histograms of surface DR3 expression in purified B cells, in resting conditions or following stimulation with anti-IgM (n = 10). Analyses were gated on lymphocytes (based on forward and side scatter), living cells (7AAD-negative), and B cells (CD19-positive). (**B**) Surface DR3 expression in resting and BCR-stimulated B cells (n = 10). Data are expressed as DR3-expression median fluorescence intensity (MFI) divided by isotype-matched control (relative median fluorescence intensity = RMFI). Comparison between resting and anti-IgM-stimulated B cells was performed by the two-sample Wilcoxon signed rank sum test. (**C**) Surface DR3 expression in IgM-negative (IgM-) and IgM-positive (IgM+) B cells (n = 10). Data are expressed as difference in DR3-expression median fluorescence intensity (MFI) divided by isotype-matched control (relative median fluorescence intensity = RMFI). Comparison between IgM-negative and IgM-positive B cells was performed by the Mann Whitney test. Data are represented as mean ± SEM. (**D**) Western blot analysis of cell lysates of purified B cells (n = 4), in resting conditions or following stimulation with anti-IgM. The level of DR3 induction after anti-IgM stimulation is reported as fold change.

To characterize DR3 expression at a protein level in human B cells, first we analyzed surface DR3 expression in resting and anti-IgM-activated B cells obtained from human PBMC samples (n = 10) by flow cytometry. B cells expressed minimal, if any, detectable levels of DR3 surface expression when they were both uncultured (time 0, data not shown) and cultured for 24 h (Fig. 1A and Fig. 1B). Following stimulation with anti-IgM, a statistically significant increase of DR3 expression (*p* = 0.002) was observed in B cells (Fig. 1A and Fig. 1B). Induced DR3 expression, measured as the fold-change in MFI between anti-IgM-stimulated and resting conditions, varied amongst the B cells, ranging from 1.6- to 13-fold change (Fig. 1B). Further, calculating the variance (σ^2^) of DR3 fluorescence intensity confirmed the high variability of DR3 expression among anti-IgM-stimulated samples (σ^2^ = 14.1). As expected, augmented DR3 expression following stimulation with anti-IgM was specifically detected in the IgM-expressing B-cell subset but not in IgM-negative B cells (*p*<0.001; Fig. 1C).

In order to confirm flow cytometry data by an independent assay, Western blot analysis was performed on B-cell total lysate extracts. DR3 exists as at least 11 isoforms generated by pre-mRNA alternative splicing. The major isoform has a molecular weight of 47 kD [28]. Accordingly, several isoforms were identified in B cells by antibodies directed against the intracellular domain of DR3 (Fig. 1D). Those included the isoform at 47 kD and an additional isoform migrating at approximately 40 kD (Fig. 1D). Consistently with the flow cytometry data, minimal, if any, DR3 levels were detected in resting B cells whereas BCR stimulation with anti-IgM antibodies induced a significant increase of all DR3 isoforms (Fig. 1D).

### Expression of DR3 in vivo

**Figure 2:**
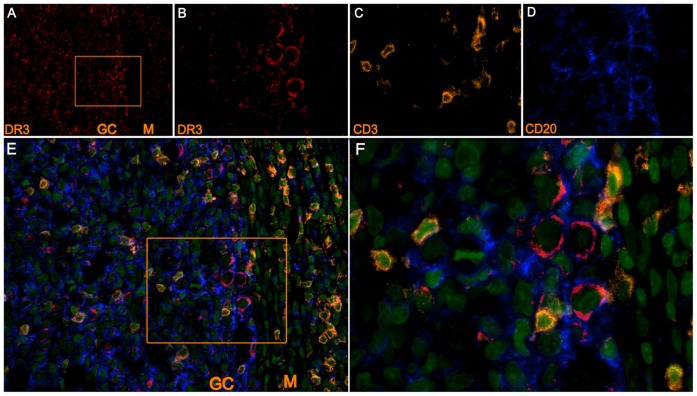
Expression of DR3 in tissue tonsil *in vivo*. Immunofluorescence analysis of DR3 in a representative tissue tonsil section (n = 4). Pseudocolour images of DR3 200x (**A**) and 1000x (**B**), CD3 (1000x) (**C**), CD20 (1000x) (**D**)**.** (**E–F**) Merged pseudocolour images of CD20 (blue), CD3 (yellow), DR3 (red) and DNA (green); (200x) (**E**), (1000x) (**F**). GC: germinal center; M: mantle zone.

To confirm the relevance of our findings, we first analyzed DR3 expression in tonsil specimens (n = 4) by using a four-color immunofluorescence approach. Figure 2A shows that tonsil germinal centers (GC) contained high numbers of cells that strongly expressed DR3 (Fig. 2B shows a magnified inset). These DR3-positive cells were of both T and B lineage, respectively identified on the basis of CD3 (Fig. 2C) or CD20 (Fig. 2D) expression, as shown by the merged pseudocolor image of DR3, CD20, CD3, and DNA (Fig. 2E and the magnified inset Fig. 2F). The highest expression of DR3 was observed in the GC centroblasts whereas intra-follicular T-cells and centrocytes showed a weak signal (Fig. 2E). In contrast, mantle-zone (M) B cells showed no detectable expression of DR3 (Fig. 2E). Because GC centroblasts are B cells activated by the antigen encounter, these data are consistent with our *in vitro* findings showing that DR3 is expressed in BCR-stimulated B cells.

**Figure 3:**
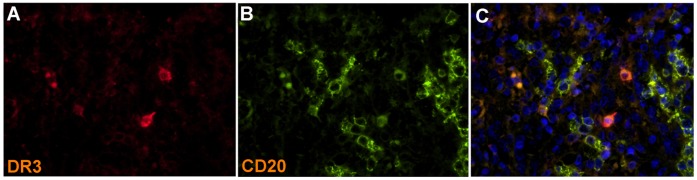
Expression of DR3 in tissue spleen *in vivo*. Immunofluorescence analysis of DR3 in a representative tissue spleen section (n = 3). Pseudocolour images of CD20 (1000x) (**A**) and DR3 (1000x) (**B**). (**C**) Merged pseudocolour image of CD20 (green), DR3 (red) and DNA (blue) (200x) (1000x).

To further confirm our findings, we analyzed DR3 expression in spleen specimens (n = 3) containing a few number of GCs (and therefore a few antigen-activated B cells) by using a four-color immunofluorescence approach. This analysis revealed the occasional presence of only a few DR3-positive cells in the spleen white pulp (Fig. 3A). These DR3-positive cells were identified as B cells on the basis of CD20 expression (Fig. 3B). Figure 3C shows the merged pseudocolor image of DR3, CD20, and DNA. These data support our *in vitro* findings showing that unstimulated B cells dot not express DR3 molecule.

### TL1A Reduces B-cell Proliferation

**Figure 4:**
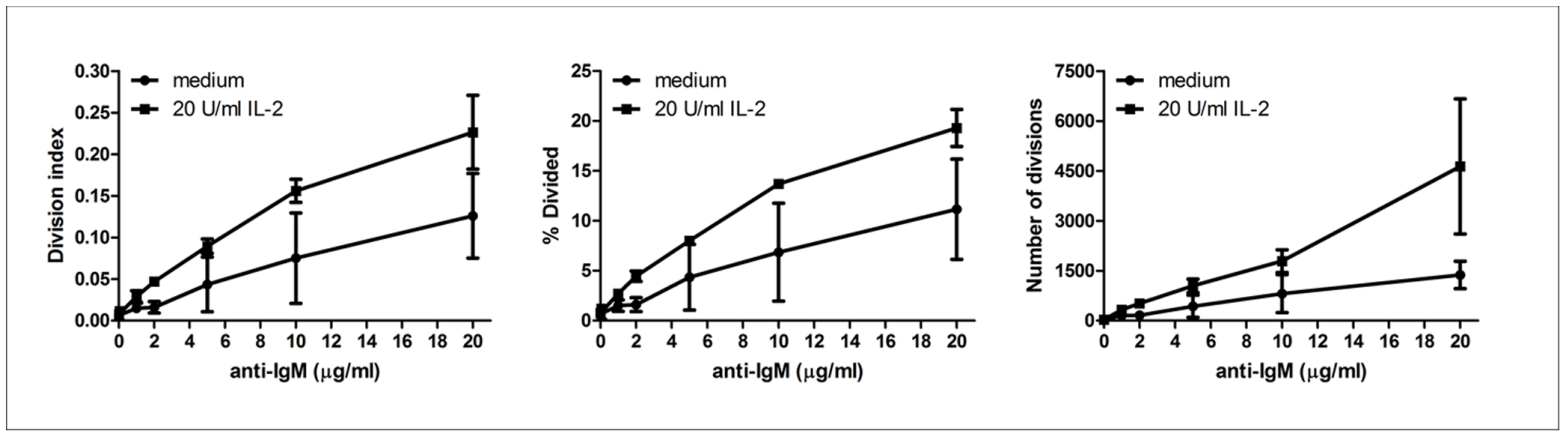
B-cell proliferation induced by increasing doses of anti-IgM antibodies in the presence of IL-2. CFSE-labeled purified B cells were activated with different doses of anti-IgM antibodies (1, 2, 5, 10, 20 µg/ml) in the presence or absence of 20 U/ml IL-2 for 96 h, and analyzed for CFSE dilution. Three parameters were calculated (division index, percentage (%) divided and number of divisions) and represented in distinct graphs.

Data on DR3 expression prompted us to investigate whether DR3 was biologically active in B cells. As it has been described that TL1A/DR3 interactions enhance cell proliferation of suboptimally activated T cells *in vitro* [12], [16], [29], we sought to investigate whether DR3 expressed on B cells could also modulate B-cell proliferative response. Therefore, B cells were incubated with different doses of anti-IgM (1, 2, 5, 10, 20 µg/ml) in the presence or absence of 20 U/ml IL-2 at different times (24, 48, 72 and 96 h). Dose-response curves indicated that, in the presence of IL-2, 2 µg/ml anti-IgM induced a suboptimal proliferative response whereas 20 µg/ml anti-IgM evoked maximal proliferative response (Fig. 4). Importantly, under these conditions, B cells expressed DR3 at any time-point analyzed, i.e. 24, 48, 72 and 96 h (data not shown). Time course curves indicated that maximal proliferative response was achieved at 96 h (data not shown). Therefore, the concentrations of 2 µg/ml or 20 µg/ml anti-IgM, 20 U/ml IL-2, in the presence or absence of 100 ng/ml human recombinant TL1A, and a 96-h time point were considered appropriate conditions to observe any eventual proliferation modulation mediated by TL1A.

**Figure 5:**
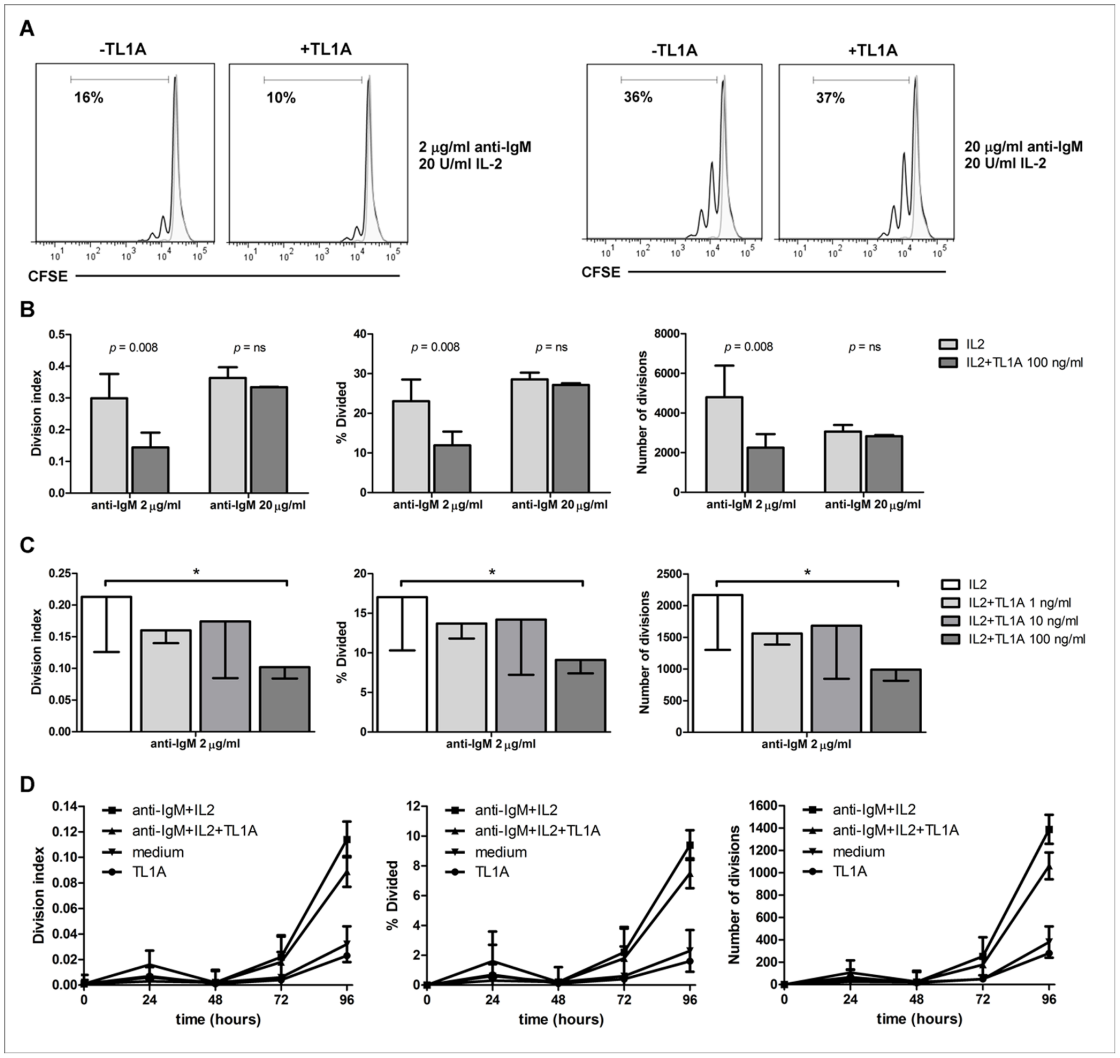
TL1A reduces proliferation of activated B-cells. (**A**) Representative flow cytometry histograms of CFSE-labeled B cells stimulated (empty curve) or not (grey curve) with 2 µg/ml (n = 8) or 20 µg/ml (n = 3) anti-IgM and 20 U/ml IL-2 for 96 h, in the presence or absence of 100 ng/ml TL1A. Bold numbers indicate percentage of proliferating cells. Analyses are gated on lymphocytes (based on forward and side scatter), and living (7AAD-negative) B cells (CD19-positive). (**B**) Proliferating B cells were stimulated with 2 µg/ml (n = 8) or 20 µg/ml (n = 3) anti-IgM and 20 U/ml IL-2 for 96 h, in the presence or absence of 100 ng/ml TL1A. Three parameters were calculated (division index, % divided and number of divisions) and represented as distinct histograms. Data are represented as mean±SEM. Comparison between treatments was performed by the two-sample Wilcoxon signed rank sum test. (**C**) Proliferation of B cells stimulated with 2 µg/ml anti-IgM, 20 U/ml IL-2 for 96 h, in the presence of different doses of TL1A (n = 3). Three parameters were calculated (division index, % divided and number of divisions) and represented as separated histograms. Data are represented as mean±SEM. * = *p*<0.05. (**D**) Time-course experiment (n = 2) of CFSE-labeled purified B cells stimulated with 2 µg/ml anti-IgM, 20 U/ml IL-2; 2 µg/ml anti-IgM, 20 U/ml IL-2, 100 ng/ml TL1A; 100 ng/ml TL1A; or medium. Three parameters were calculated (division index, % divided and number of divisions) and represented as separated graphs.

Remarkably, in contrast to its effects on T-cell proliferation [13], [16], [29], TL1A significantly decreased proliferation of B cells activated with suboptimal doses of anti-IgM (*p* = 0.008) whereas did not affect B-cell proliferation induced by saturating doses of anti-IgM (Fig. 5A and Fig. 5B). Dose-response studies demonstrated that maximal response in modulating B-cell proliferation was achieved with 100 ng/ml TL1A (*p* = 0.015) (Fig. 5C). In addition, time-course experiments revealed that the ability of TL1A to modulate B-cell proliferation was observed at the same time to the onset of proliferative activity following stimulation with anti-IgM and IL-2 (72 h) and remained up to 96 h of incubation (Fig. 5D). No effect was observed on cell proliferation when B cells were incubated with TL1A alone, in the absence of anti-IgM and IL-2 (Fig. 5D).

**Figure 6:**
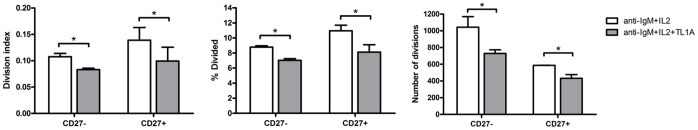
TL1A induces similar reduction extents of proliferation in the CD27+ and CD27− B-cell subsets. CFSE-labeled purified B cells (n = 2) were activated with 2 µg/ml anti IgM, 20 U/ml IL-2 for 96 h, in presence or absence of 100 ng/ml TL1A. Analyses were gated on lymphocytes (based on forward and side scatter), living (7AAD-negative) and CD19-positive cells and discriminating CD27+ and CD27− cells. Three parameters were calculated (division index, % divided and number of divisions) and represented as separated histograms. Data are represented as mean±SEM. * = *p*<0.05.

**Figure 7:**
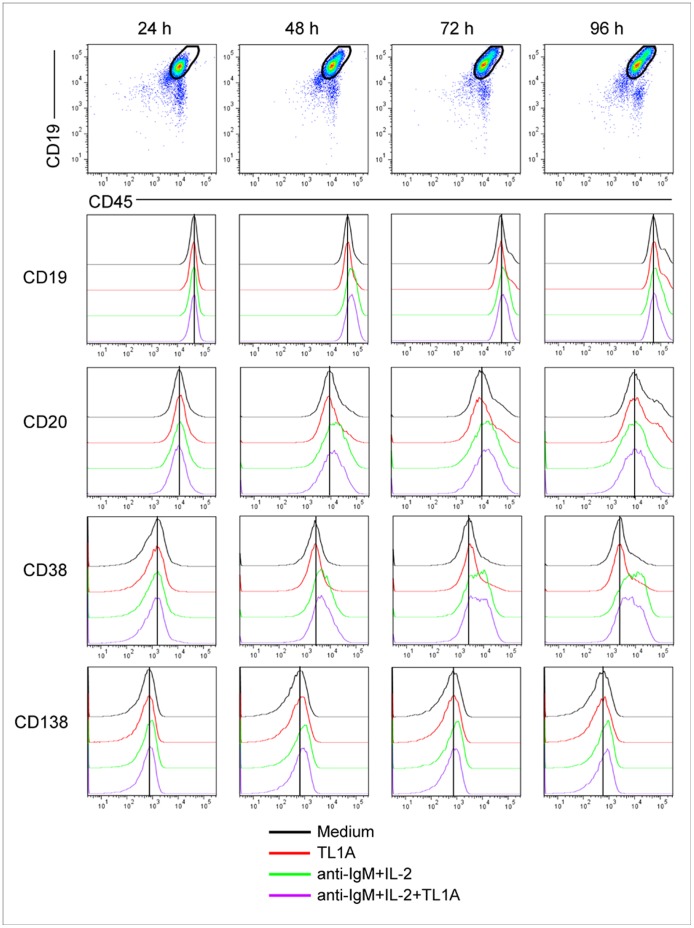
TL1A does not modulate surface expression of CD19, CD20, CD38, and CD138 in B cells. Data from a representative experiment (n = 2) on CD19, CD20, CD38, and CD138 expression in B cells activated with 2 µg/ml anti-IgM, 20 U/ml IL-2 in the presence or absence of 100 ng/ml TL1A at different time point (24, 48, 72, 96 h).

Next, we explored whether the TL1A-mediated inhibitory effects on B-cell proliferation could differentially affect memory (CD27+) and naïve (CD27−) B cells. As shown in Fig. 6, recombinant TL1A induced similar extents of proliferation reduction in the two B-cell subsets. Cell-proliferation reduction was not paralleled by significant changes in CD19, CD20, CD38 and CD138 expression, as detected by flow cytometry analysis of B cells activated with anti-IgM and IL-2 at different time points upon TL1A treatment (24, 48, 72, 96 h) (Fig. 7).

**Figure 8:**
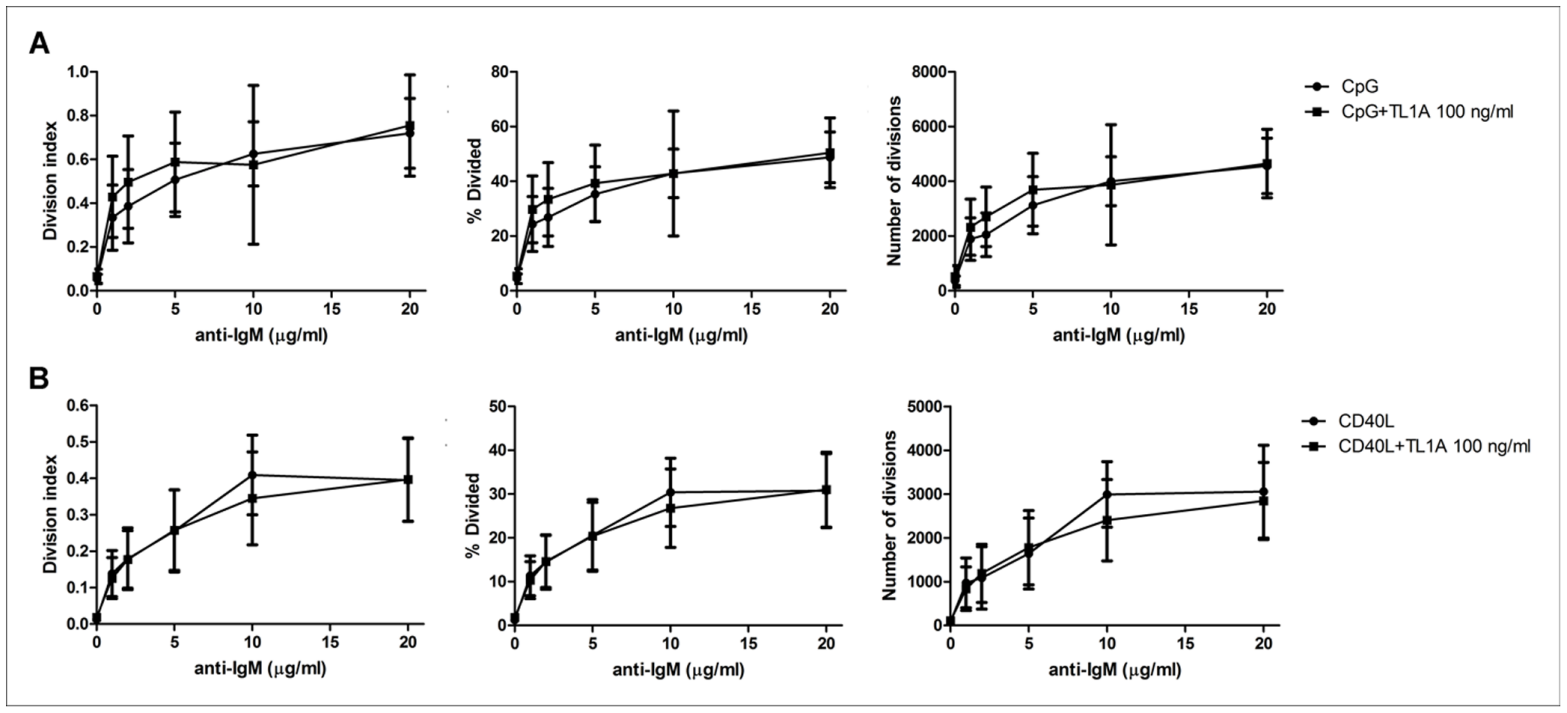
TL1A does not modulate B-cell proliferation induced by anti-IgM and CpG-ODN or CD40L. (**A**) Proliferating B cells were stimulated with different doses of anti-IgM antibodies (1, 2, 5, 10, 20 µg/ml) and of 2.5 µg/ml CpG-ODN for 96 h, in the presence or absence of 100 ng/ml TL1A (n = 4). Three parameters were calculated (division index, % divided and number of divisions) and represented as separated graphs. (**B**) Proliferating B cells were stimulated with different doses of anti-IgM antibodies (1, 2, 5, 10, 20 µg/ml) in the presence of 50 ng/ml CD40L for 96 h, in the presence or absence of 100 ng/ml TL1A (n = 4). Three parameters were calculated (division index, % divided and number of divisions) and represented as separated graphs.

To assess the specificity of TL1A effect to B cell proliferative stimuli, B cells were induced to proliferate by treatment with different doses of anti-IgM in conjunction with CpG-ODN or CD40L, in the presence or absence of TL1A. Figure 8 shows that TL1A did not affect B cell proliferation induced by either anti-IgM+CpG-ODN or anti-IgM+CD40L, at any concentration of anti-IgM (range 1–20 µg/ml). CpG-ODN or CD40L in the absence of anti-IgM did not induce B-cell proliferation (Fig. 8).

### TL1A does not Affect B-cell Survival

**Figure 9:**
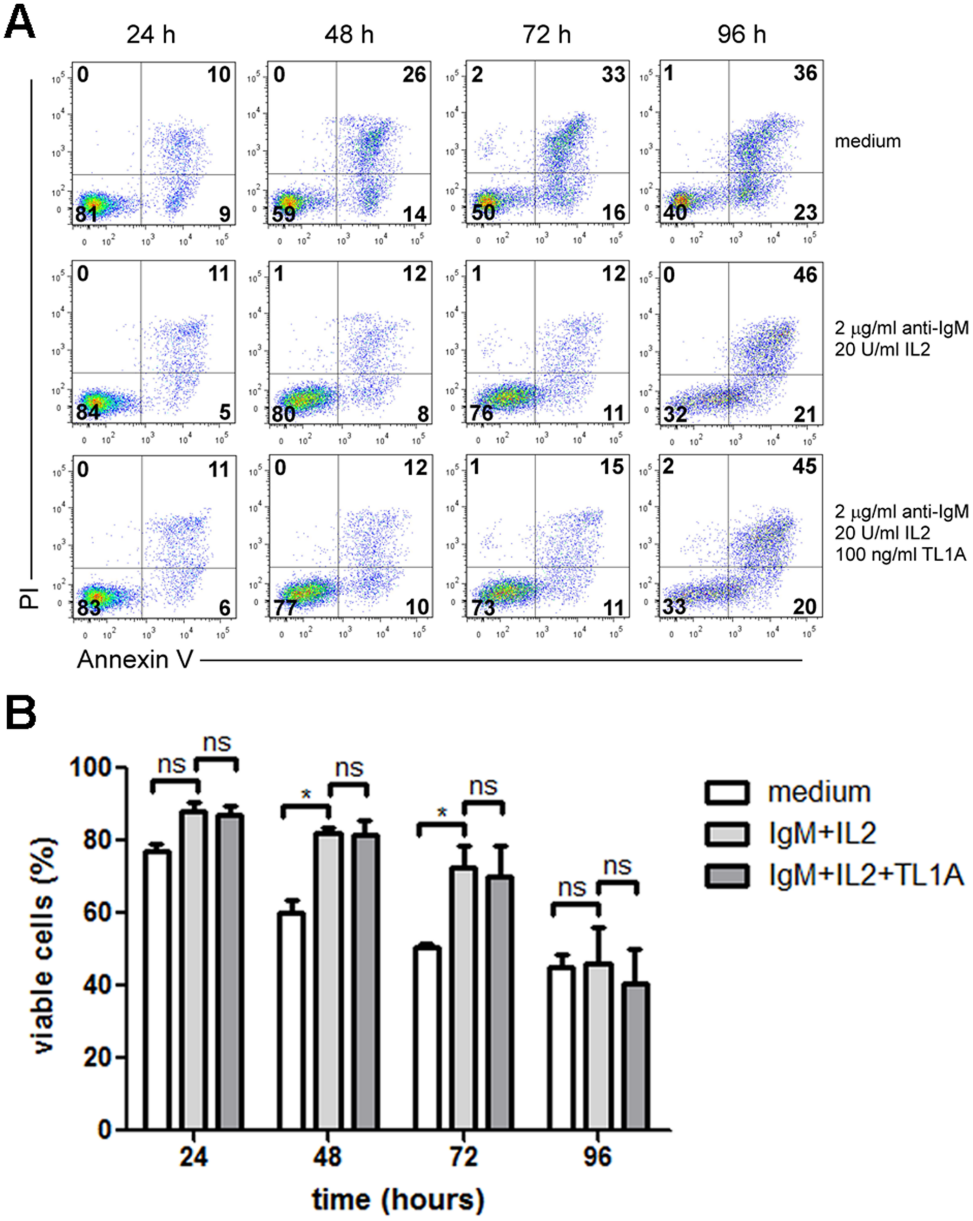
TL1A does not affect B-cell survival. Purified B cells were activated with 2 µg/ml anti-IgM, 20 U/ml IL-2 for 24, 48,72 and 96 h, in presence or absence of 100 ng/ml TL1A, stained with annexinV/PI and analyzed by flow cytometry. Analyses were gated on CD19-positive cells. (**A**) Dot plots representatives of three independent experiments. Bold numbers indicate percentage of cells in each quadrant. (**B**) Percentage of viable cells (annexinV-negative/PI-negative) following incubation with 2 µg/ml anti-IgM, 20 U/ml IL-2; 2 µg/ml anti-IgM, 20 U/ml IL-2, 100 ng/ml TL1A; medium. Data are represented as mean±SEM. * = *p*<0.05; ns = not significant.

Depending on the cellular context in which DR3 is triggered, its activation can result in induction of apoptosis [5], [30]. This raised the possibility that the reduced CFSE dilution induced in B cells by TL1A was due to reduced survival. To test this, purified B cells were incubated with anti-IgM and IL-2 in the presence or absence of recombinant TL1A and cell survival was measured by annexinV/PI staining and flow cytometry at 24, 48, 72 and 96 h following TL1A treatment. As expected, stimulation with anti-IgM, IL-2 protected B cells from spontaneous apoptosis whereas no significant changes in cell viability were observed in anti-IgM/IL-2 activated B cells treated with TL1A (Fig. 9), thus demonstrating that reduced CFSE dilution induced in B cells by TL1A was due to decreased cell division and not to reduced survival.

## Discussion

DR3 2,6 has been shown to modulate the functions of T cells, NK and NKT cells [6], [11], [16], [17], [21], [22]. B cells express very little DR3 full-length mRNA whereas they express combination of the other isoforms, encoding potentially secreted molecules [5]. However, DR3 protein expression and its effects on B cells remain largely unknown. In this study, we describe for the first time that B cells from human blood express significant amounts of DR3 in response to the polyclonal stimulation of BCR. The relevance of these results has been confirmed by immunofluorescence analysis of tonsil and spleen tissue specimens, which show that antigen-stimulated B cells (centroblasts) in tonsil GC express high levels of DR3 whereas a few DR3-positive B cells are detectable in the white pulp of spleens showing rare GC.

Remarkably, we demonstrated that TL1A reduces B-cell proliferation induced by anti-IgM-antibodies and IL-2. This result is opposite of that observed in T cells, where TL1A/DR3 interactions costimulate proliferation of suboptimally activated cells [13], [16], [29]. However, consistently with TL1A effects on T cells, our data support the notion that TL1A acts as a cell modulator that cannot function without antigenic activation signals.

TL1A-induced inhibition of B-cell proliferation appears to be independent of apoptotic mechanisms since TL1A does not trigger cell death in activated B cells. This is consistent with previous studies showing that TL1A induces apoptosis in overexpression cell systems and in DR3-expressing cell lines, whereas primary T cells do not undergo apoptosis when treated with TL1A [7].

In this study, we used peripheral blood B cells, which consist of naïve and memory B cells. We show that the extent of proliferation inhibition induced by TL1A is similar in the two B-cell subsets, in contrast to what observed in T cells, in which the effects mediated by TL1A are more pronounced in memory versus naïve T cells [29], [31]. Further, TL1A-mediated inhibition of proliferation is not paralleled by changes in B-cell phenotype, thus indicating that TL1A does not affect B-cell differentiation.

Although changes in IgM expression induced by TL1A could not be evaluated in this experimental condition (the presence of anti-IgM induces internalization of IgM molecules, irrespectively of the presence of TL1A), TL1A does not affect IgD and IgG surface expression on B cells activated with anti-IgM and IL-2 (data not shown). These data, although partial, are in agreement with previous data indicating a normal antibody production in the absence of DR3, in murine models of experimental autoimmune encephalomyelitis (EAE) [16] and experimental antigen-induced arthritis (AIA) [32].

TL1A-mediated inhibition of proliferation is specific to anti-IgM and IL-2 stimuli, since TL1A does not affect proliferation induced by other B-cell specific stimuli, such as anti-IgM in conjunction with CpG-ODN or CD40 ligand. This finding suggests that *in vivo* TL1A can modulate B-cell proliferation in a context conditioned by the presence of IL-2.

The mechanisms underlying TL1A inhibitory effects on B cells are unclear yet; however, the finding that TL1A reduces proliferation of purified B cells rules out that other cells can indirectly mediate this effect. TL1A is mainly expressed by macrophages, dendritic cells, endothelial cells, and T cells activated by inflammatory stimuli [7], [11]. In contrast, B cells are unable to produce TL1A, as assessed by immunofluorescence and ELISA analyses, both in resting conditions and upon activation with anti-IgM (data not shown). Therefore, several cell types can produce TL1A cytokine potentially acting on B cells in various physiological and pathological settings, whilst the existence of an autocrine production of TL1A can be reasonably excluded.

Herein, we show that B-cell proliferation is inhibited by TL1A *in vitro* and one may speculate a similar effect *in vivo*, where it may have relevant implications in both physiological and pathological immune responses. TL1A costimulates T-cell proliferation and cytokine production of activated T cells *in vitro* [11], [12], thus defining a role for TL1A as a T-cell costimulator. In addition, TL1A biases T-cell differentiation toward Th1 and Th17 T cells [12], [13], [17] and modulate Treg expansion and functions [18]–[20], therefore suggesting a role in regulating the adaptive immune response. In pathological settings, TL1A interactions with DR3-expressing T cells have been shown to play a crucial role in driving inflammatory processes at the site of inflammation in several T-cell-dependent autoimmune disease models, such as rheumatoid arthritis (RA) and inflammatory bowel disease (IBD) [16], [25], [26], [31], [33]–[34]. The effects of TL1A described in T cells, both *in vitro* and in animals models of autoimmune diseases may be apparently in contrast to the inhibitory effects mediated by TL1A on B-cell proliferation herein described. However, it has been shown that effects of TL1A on T-cell differentiation *in vitro* are largely dependent on experimental settings. For example, exogenous TL1A suppresses the ability of human CD4+ T cells to differentiate into Th17 in the presence of IL-2 [17], [29] whereas promotes Th17 fate when IL-2 is limiting [17]. Also, TGF-β-induced T-cell differentiation into Treg is inhibited by TL1A when IL-2 is not limiting whereas TL1A does not suppress and can even enhance Treg development when IL-2 is not added [26], [29], [35]. Therefore, TL1A homeostatic functions seem to be highly dependent on the context of the immune response that is being modulated. Accordingly, the inhibitory effects of TL1A on B-cell proliferation may also depend on the specific experimental setting *in vitro*. Similarly, *in vivo* TL1A may be able to limit B-cell expansion in a particular condition of the immune response.

This study shows that TL1A is able to inhibit B-cell proliferation *in vitro* and suggests that TL1A may contribute to homeostasis of effector B-cell functions in immune response and host defense. Together with previous data from the literature, these results support an important role for TL1A in modulating the cell-mediated immune responses.
